# I Am More than HIV: A Qualitative Exploration of Factors That Can Strengthen Resilience Among HIV-Positive Gay Men in Australia

**DOI:** 10.1007/s13178-023-00829-9

**Published:** 2023-06-10

**Authors:** Neil A. Lucas, Glen W. Bates, Simone Buzwell

**Affiliations:** Department of Psychology, School of Health Sciences, Swinburne University of Technology, John Street, Hawthorn, 3122 Melbourne, Australia

**Keywords:** HIV, Gay, Men, Stigma, Resilience, Qualitative

## Abstract

**Introduction:**

HIV-positive gay men continue to experience stigma related to sexual orientation and HIV status. Although resilience toward such stressors can be achieved, limited Australian research exists that examines how resilience is strengthened toward these dual stigmas.

**Methods:**

A total of 20 men from Melbourne, Australia, participated in semi-structured interviews between March and October 2019 to explore ways in which they manage such stigmas.

**Results:**

Reflexive thematic analysis identified two primary themes: (1) “intrapersonal control,” which relates to individual mind set and lifestyle changes that participants utilized to strengthen resilience; (2) “systemic change,” which includes participants’ needs for better public health messaging. Findings show resilience was enhanced when proactive approaches to sexual orientation, HIV health appraisal, lifestyle changes, and social support were made. Further, outdated HIV awareness campaigns and a lack of current messaging regarding HIV transmission in the wider community were identified as inhibiting resilience development and promoting stigma among gay men.

**Conclusion:**

The results from this study show ways that Australian gay men strengthen their resilience through both intrapersonal (e.g., self-awareness, reappraisal, and self-efficacy) and external resources (e.g., education and public awareness) and how health care providers and social policy makers could better support the men to achieve this.

**Social-Policy Implications:**

Findings suggest that targeted public health responses are required to compliment the advances made in biomedicine and viral suppression.

## Introduction

For many gay men with human immunodeficiency virus (HIV), the dual stigmas of sexual orientation and HIV status can adversely affect their psychological wellbeing and encourage maladaptive coping behaviors (Blanch et al., 2002; Cama et al., 2020). Since the start of the HIV pandemic in the early 1980s, gay men have been associated with HIV infection which has stigmatized this community through the messaging that HIV is a “gay man’s disease” (US Department of Health & Human Services, 2018). Nearly 40 years later, associations persist between gay men and HIV, not only in the general population, but within the gay community itself, creating a divide between HIV-negative and HIV-positive gay men (Smit et al., 2012). Such stigmatization continues despite advancements in HIV biomedicine with HIV-positive gay men continuing to face rejection and ostracization, which hamper their physical and mental health (Reinius et al., 2021).

Many individuals within the gay community also continue to report internalized stigma (e.g., emotional absorption of negative stereotypes related to their sexual orientation) and enacted stigma (e.g., discriminatory and prejudiced behavior perpetuated by heterosexist attitudes), notwithstanding advancements in laws that aim to protect individuals from inequality (Ayhan et al., 2020; Goldenberg et al., 2018; Meyer, 2016). Research demonstrates the negative impact sexual orientation stigma has had on mental and physical health (Fingerhut et al., 2010; Mallory et al., 2015; Skerrett et al., 2015) and, for gay men living with HIV, the stigma is amplified. This can result in a reluctance to attend health-related services, putting both the individual and the wider community at risk.

Not all HIV-positive gay men experience stigma in the same way and many employ varied coping strategies and ways of processing stigma experiences (Arístegui et al., 2018). In Australia, there is only limited research on how gay men process stigmatized identities and how they manage stigma and strengthen resilience post HIV diagnosis. The purpose of the current investigation was to explore the lived experiences of sexual orientation and HIV-related stigmas of Australian gay men and to investigate the strategies they have used to strengthen their resilience.

### Sexual Orientation Related Stigma

A leading paradigm in sexual minority stigma research is Meyer’s (1995) minority stress model. Meyer posits that social conditions characterized by prejudice toward a minority group (i.e., heterosexism or homophobia) can predispose individuals to experience stress related to their minority status (Meyer, 1995; Peleg & Hartman, 2019). For some individuals, the impact of identifying with a minority sexual orientation increases their expectations of rejection, internalized homophobia, or motivations to conceal sexual identity (Meyer, 1995; Walch et al., 2016). It is well established that sexual orientation related stigma negatively affects psychological wellbeing and increases suicidal ideation in lesbian, gay, bisexual, transgender, intersex, and asexual (LGBTQIA+) individuals (Herek et al., 2015; Lyons et al., 2022; Meyer, 1995; Pachankis et al., 2020; Skerrett et al., 2015). As a result, many LGBTQIA+ individuals require a disproportionately greater amount of mental healthcare compared to their heterosexual counterparts (Hatzenbuehler & Pachankis, 2016; Lea et al., 2014; Pachankis, 2014).

Unfortunately, LGBTQIA+ health care needs are difficult to assess due to factors linked to minority status. For example, the fear of discrimination from healthcare providers can deter individuals from seeking health treatment leaving vulnerable individuals at risk (Pachankis, 2014). Moreover, seeking support for sexual identity issues may not be an option due to the limited number of LGBTQIA+ inclusive providers, or fear of “being outed” if an inclusive provider is available. This experience of fear related to third-party disclosure is exacerbated within rural or regional areas (Hubach et al., 2015; Lyons et al., 2015; Morandini et al., 2015). The impact of a lack of mental health support can contribute to low psychological wellbeing and has been found to result in maladaptive coping strategies including alcohol and drug misuse or risky sexual behavior (Lelutiu-Weinberger et al., 2013).

Although some Western countries have recently shown greater acceptance of non-heterosexual sexual orientations (Bränström & Pachankis, 2019; Riffkin, 2014), including implementation of legislation regarding LGBTQIA+ equality and rights (Hooghe & Meeusen, 2013), sexual orientation-related stigma and sexual minority stress continue (Cronin et al., 2021). For example, when the Australian Federal Government conducted a non-binding voluntary postal survey in 2017 to gage support for marriage equality, many LGBTQIA+ individuals reported psychological distress and perceived stigma due to negative media messaging and public scrutiny of their sexual orientation and right to marry (Casey et al., 2020). These effects continued even after the YES vote was carried (Ecker et al., 2019; Verrelli et al., 2019). Unfortunately, many LGBTQIA+ individuals still live with daily challenges of overt prejudice and subtle microaggressions, regardless of equality policies and anti-discrimination laws (Livingston et al., 2020). Thus, such discrepancies between policy and lived experience exist, and this has had a negative effect on the psychological wellbeing for many LGBTQIA+ individuals.

### HIV-Related Stigma

In 2020, it was estimated that 29,090 Australians were living with HIV (0.14% population). Of this number, an estimated 21,550 are thought to have contracted HIV through male-to-male sexual contact, for example, gay, bisexual, and other men who have sex with men (GBMSM) (Kirby Institute, 2020). Although there has been a decrease of 45% of HIV transmission notifications among GBMSM between in the last five years, approximately 74% of HIV cases in Australia remain concentrated among this population (Kirby Institute, 2020). As such, it is not only sexual orientation stigma that negatively impacts psychological wellbeing, but the combined impact of both sexual orientation and HIV-related stigmas can be difficult to navigate for this population (Iott et al., 2022; Skerrett et al., 2015).

Improvements in biomedicine might be expected to impact HIV-related stigma. Since 1997, effective antiretroviral therapy (ART) has been available changing HIV from a terminal to a chronic health condition (Deeks et al., 2013). In Australia, it is estimated that approximately 96% of HIV-positive GBMSM are currently under medical management and receiving combination ART, resulting in lower transmission rates and an undetectable viral load for many men (Kirby Institute, 2020). Indeed, the concept of viral suppression through ART adherence has been examined since the late-2000s and promoted as “undetectable = untransmittable” (U = U) (Eisinger et al., 2019). The U = U campaign illustrates how HIV-positive individuals who adhere to their ART regime can suppress their viral load to an undetectable level leading to a negligible risk of HIV transmission (Cambiano et al., 2019). However, even with such advancements in biomedicine and many men living with an undetectable suppressed viral load, HIV-positive gay men still face stigma related to sexual orientation and HIV status, such as health care provider discrimination (Algarin et al., 2020) and social rejection due to their chronic health condition (Roth et al., 2022).

Drawing parallels with the minority stress paradigm (Meyer, 1995, 2003), many gay men living with HIV face rejection, internalized stigma, or concealment motivations related to their HIV status (Power et al., 2021). Research has shown that concealment of HIV status reduces access to social support, which can negatively affect both mental and physical health (Walker, 2019; Ziersch et al., 2021). Further, for some individuals, fear of the stigma associated with their HIV status results in missed medical appointments, less viral load testing and caution related to accessing HIV medication due to fear of public disclosure (Lea et al., 2019; Qiao et al., 2018). This can contribute to further health complications, as well as potentially increasing the risk of HIV transmission if the individual engages in risky sexual behavior (Brown et al., 2017; Mey et al., 2017). Thus, the stigma associated with accessing HIV-related health care can have an impact on transmission and infection rates in the wider community.

### Resilience Toward Sexual Orientation and HIV-Related Stigma

Not all gay men with HIV experience poor mental health, with some men demonstrating resilience toward minority status stigma (Asakura & Craig, 2014; Lyons et al., 2016). Resilience is a broad and emerging area of research; however, it can generally be defined as a dynamic process by which individuals utilize protective factors and resources to their benefit (Stainton et al., 2019). Depending on the level of analysis, resilience has been examined as a trait, process, or resource and comprises both interpersonal and environmental components (De Santis et al., 2013; Harper et al., 2014).

Much international HIV stigma research over the past decade has sought to quantify resilience through scores on self-report scales that measure the individual, interpersonal, or community-level resources that individuals might utilize to strengthen resilience (Brewer et al., 2020; Dulin et al., 2018; Gottert et al., 2019; Hussen et al., 2017). While these studies demonstrate that individuals who have access to such resources report higher resilience scores, these quantitative measures do not capture the multifaceted construct of stigma experience (e.g., multiple adversities or access to HIV-support services or LGBTQIA+ inclusive providers) that impacts on resilience development as evident in qualitative research findings (Dulin et al., 2018; Emlet et al., 2011; Harper et al., 2014; Liboro et al., 2021a, b). Thus, the need to understand these complex processes invites research on the multifaceted nature of stigma, for example, via applying intersectionality theory (Cole, 2009) to stigma research. Intersectionality theory considers the overlap of intersecting identities (e.g., race, ethnicity, sexual orientation, and socioeconomic status to name a few) that may influence stigma experiences and therefore impact on resilience development across the lifespan. Indeed, many stigma researchers are adopting intersectionality theory as an approach when examining manifestations of stigmatization from multiple sources (Turan et al., 2019), which is pertinent when exploring the impact of HIV and the interlocking inequality experiences of stigmatized sexual orientation. Importantly, stigma research utilizing intersectionality theory has shown that examining how multiple stigmas interact on stigma experience improves the understanding of how to best support individuals experiencing stigma (Earnshaw et al., 2022). This illustrates the necessity of further research into how stigmas impact vulnerable minority populations.

In Australia, where HIV infection is still concentrated among GBMSM, resilience development among this population is a young and emerging area of research. A quantitative study by Lyons et al. (2016) showed Australian men who scored lower on internalized HIV stigma measures reported higher resilience scores, suggesting that resilience-training programs aimed at HIV-positive gay men may reduce the impact of internalized HIV-related stigma. Similarly, another quantitative study (Lyons & Heywood, 2016) explored collective resilience as a protective factor for mental health among HIV-positive gay men. The authors found that men, who were connected to groups or communities with high levels of resilience, reported lower scores on mental health symptoms and internalized-stigma. These two studies demonstrate that Australian HIV-positive gay men can indeed increase their resilience. However, the individual strategies employed by Australian men to achieve resilience to such stigmas are yet to be identified and warrant further investigation.

### The Present Study

The present study involved a qualitative examination of how Australian HIV-positive gay men may strengthen resilience toward sexual orientation and HIV stigmas. The aim of this study was to explore the practical ways in which Australian men respond to stigma and to elicit themes that can help promote better health management strategies among gay men struggling with their HIV diagnosis and/or sexual orientation. As such, the following research question was posed: What factors strengthen resilience toward sexual orientation and HIV-related stigmas among Australian gay men?

## Methods

### Design

The study was situated within an interpretivist and constructivist paradigm to elicit participants’ attitudes, opinions, and understanding of personal lived experience of dual stigmas. A reflexive inductive thematic analysis (TA) was chosen consistent with guidelines set by Braun and Clarke (2020). Reflexive TA was used as it is flexible, diverse, and data driven with coding and theme development directed by the content of the data and not presupposed. Within this six-phased analytic approach, each theme builds on the previous phase in a recursive process (see "Reflexive Thematic Analysis" for steps followed).

### Procedure and Recruitment

Prior to commencement, the study received ethical approval (SHR2018/388). The participants were recruited via a series of advertisements placed at a Melbourne-based HIV service provider (where a medical HIV diagnosis is required to access the service). Participants were eligible if they (1) self-identified as gay and (2) self-identified as male, (3) had a medical diagnosis of HIV, (4) were over 18 years of age, (5) lived in Australia, (6) had reported experiencing stigma related to sexual orientation and HIV status, and (7) could complete the interview in English. Each participant was given information about the study via email or hardcopy and gave verbal and written consent prior to interview commencement. Participants were pre-screened via telephone regarding each of the inclusion criteria prior to interview.

#### Participants

Twenty-nine gay men volunteered to be interviewed and were pre-screened. Of these, nine did not wish to be interviewed after being provided information about the study, with a total of 20 gay men completing interviews. One interview was completed via telephone with the remaining 19 interviews completed face-to-face. Participants were aged between 33 and 67 years, with a mean age of 52.85 years (SD = 9.29). Time since HIV diagnosis ranged between six and 36 years, with an average of 16.20 years (SD = 9.53). Further participant characteristics are provided in Table 1.

**Table 1:** Characteristics of the sample (*N* = 20)

	Total *n*
Sexual orientation Gay	20 (100%)
Ethnicity White Australian/Anglo/European	20 (100%)
Born in Australia	18 (90%)
Born overseas	2 (10%)
Age by ABS grouping categories^*^	
Middle adults (25–44 years) Older adults (45–64 years) Retirement (65 + years)	5 (25%) 13 (65%) 2 (10%)
Education	
Year 10 high school Year 12 high school Diploma Bachelor’s degree Postgraduate degree	8 (40%) 3 (15%) 4 (20%) 3 (15%) 2 (10%)
Income	
Full time employment Part time employment Unemployed/disability Student Retired	1 (5%) 0 (0%) 14 (70%) 2 (10%) 3 (15%)
Antiretroviral medication	
Yes No	20 (100%) 0 (0%)
Undetectable viral load	
Yes No Unsure	20 (100%) 0 (0%) 0 (0%)

*Age groups determined by the Australian Bureau of Statistics (2014)

Characteristics of sample show that all participants identified as white/Anglo-Saxon with 60% of participants having completed formal education past year 10. Only one participant was employed full time, with 70% of the sample reporting unemployment or disability. All participants were virally suppressed and adhered to a medication regime and regular medical appointments. The ages of participants were grouped into three categories, middle-adults, older-adults, and retirement-age, with most participants (65%) classed as being of older-adult age. Age groupings were categorized as per guidelines set out by Australian Bureau of Statistics (2014).

### Interview

An interview schedule was created by the current researchers who have experience in working with sexual minority populations (see "Research Team"). Interview questions were generated, reviewed by two authors, and piloted with colleagues and peers with experience in qualitative research and the research area and revised until consensus was met. The final interview schedule was developed to explore participants’ experiences of both sexual orientation and HIV stigma and responses to developing resilience (see Table 2).

**Table 2:** Key interview questions related to stigma and resilience

Item	Question
1.	Can you tell me about a time when you have felt stigmatized due to sexual orientation or HIV status and how did you respond?
2.	How has stigma impacted your life?
3.	What has helped you cope/build resilience toward stigma?
4.	What advice would you give to a person struggling with sexual orientation/HIV stigma or a new HIV diagnosis?
5.	Has your experience of stigma changed since you were first diagnosed? How?
6.	Is there a difference between sexual orientation and HIV stigma? How?

Participants engaged in a semi-structured interview with the first author between March and October 2019. All face-to-face interviews were conducted at an HIV support and drop-in center. Each participant completed a one-to-one semi-structured qualitative interview, with interviews ranging from 60 to 90 min (*M* = 78.80 min, SD = 4.91). Participants provided demographic information and were then asked to describe their experiences of sexual orientation and HIV stigmas and how they have responded to stigma experiences. All interviews were digitally recorded, and participants were compensated with a $50 shopping voucher for their time.

### Reflexive Thematic Analysis

The de-identified recordings were transcribed verbatim by a third-party transcription service with the first author checking each transcription against the audio recordings for accuracy. Corrections were made to transcripts where necessary. The final transcripts were uploaded to NVivo (version 12), and participant responses to each of the key resilience-based questions were extracted.

The analysis followed the six core principles of reflexive thematic analysis (Braun & Clarke, 2020). In the first phase, the first author familiarized himself with the data which involved multiple reads of each transcript to become immersed in the data content. For the second phase, the first author coded data using “labels,” of which each label elicited at least one (if not more) important observation or facet related to the research question. The third phase focussed on initial theme and sub-theme generation which was completed by the first author and involved examining the labels to identify broader patterns of meaning as well as reviewing the viability of each theme. In the fourth phase, the first and third author reviewed the themes by checking each one against the dataset to ensure each theme relayed a multi-faceted interpretation of the data and was aligned to the research question. Any discrepancies among authors related to themes (or labels) were discussed and revisions considered accordingly by the first author in a recursive process. In the fifth phase, finalization of the theme and subtheme names and definitions was completed by the first author in consultation with the third author. A review and analysis of the theme names and meanings was completed to ensure each theme relayed an accurate representation of the participants’ stories. In the sixth phase, the final write-up of the analysis of each theme and subtheme was completed by the first author and reviewed by the second and third authors. Data findings were deemed as patterns of shared meaning related to the research question.

### Research Team

The primary investigator, a gay cis-male PhD candidate and provisional psychologist, has over fifteen years of conducting group and one-on-one emotional-support across several HIV and LGBTQIA+ organizations. The second author, a cis-heterosexual male and third author, a cis-heterosexual female, have both researched, taught, and/or worked clinically with LGBTQIA+ /HIV populations and have over 64 years combined experience in academia, qualitative research, and publication. The combined and different knowledge and experience of the authors in this study allowed for collaborative discussion and reflexive engagement with the data.

## Results

Sexual orientation stigma was experienced among and reported by all participants which influenced both disclosure and concealment related to HIV diagnosis in later years. All participants had “come out” as gay prior to HIV diagnosis to at least one person. Further, all participants had experienced at least one episode of HIV-related stigma, with participants reporting both sexual orientation and HIV stigmas impacting negatively on mental health outcomes.

### Sexual Orientation Stigma and the Interaction with HIV Stigma

All men in this study expressed how sexual orientation stigma is still experienced within Australian society, despite legislative changes, including those related to same-sex marriage implemented in 2017. For example, “there’s always a sense of not quite fitting in…I often hear groups of straight guys call each other “poofter” or “queer” as a joke, but it still makes me feel uncomfortable like I’m gonna get bashed” (43 years old, six years since diagnosis). Indeed, perceptions of sexual orientation stigmatization clearly persist, with participants expressing how even with increased societal acceptance related to diverse sexual orientations, systemic homophobia continues to contribute to feelings of unease and causes concern related to personal safety. Similarly, internalized homophobia was experienced by all the men in this study, especially those aged 40 years and above (*n* = 18). Men in this study discussed how both enacted stigma and internalized stigma related to sexual orientation resulted in isolation and low self-worth and impacted on decision to disclose HIV status. Participants expressed how differences in societal acceptance of homosexuality during the last three decades has influenced stigma experience. For example:

I came out [as gay] to friends a long time ago, before AIDS started, when “gay” was still not publicly spoken about. I was living with my partner at the time. My parents never asked me about it, they just called him my flatmate. We never spoke about it. It was taboo. Even today I don’t really discuss it. My parents are long gone, but I still have cousins who don’t like it. They react badly to me. Typical Aussie rednecks. They still make me feel like shit (67 years old, 15 years since diagnosis).

For this participant, even after being “out” for close to fifty years, he continued to experience negative thoughts related to his sexual orientation due to his family’s non-acceptance. Although this participant had distanced himself from most family members, he remained cautious about seeing them within the community and commented that he would never tell them he was HIV-positive based on their reaction toward his sexual orientation.

Similarly, due to family rejection and a bad experience when coming out as gay, another participant commented how his experience of sexual orientation stigma has influenced his decision to conceal his HIV status, for example, “I am so nervous about being seen coming in here [to the HIV service] that I’ve lied before and said I’m volunteering at the service, I’m not a client” (40 years old, 10 years since diagnosis). This participant expressed shame related to his HIV status which was exacerbated by his previous negative experiences of sexual orientation stigma.

The impact of internalized homophobia on psychological distress and HIV status was also evident from another participant who commented:

I addressed my sexual orientation much later in life as being gay was a bad thing when I was younger. I even went to the doctor to see if there were pills I could get that would suppress my libido. If I didn’t want sex with men, then maybe I wasn’t gay. He told me “no” and that I should accept it. I got HIV a few years later. I still find it hard to accept being gay and HIV-positive because I chose to have gay sex (59 years old, seven years since diagnosis).

This participant discussed how his “decision” to be a gay man after not being able to suppress his libido resulted in self-blame for his HIV diagnosis, as “allowing” himself to be gay resulted in HIV infection. Although this participant disclosed he is now learning to accept himself (e.g., a gay man) and his HIV diagnosis, a part of him still feels conditioned to believe his sexual orientation is responsible for his HIV status, a clear demonstration of internalization of societal narratives that link sexual orientation to HIV infection.

In contrast to older middle-aged, older-adults, and retirement-aged adults, a younger middle-aged participant shared his views on the differences between sexual orientation and HIV-related stigmas. He explained:

There were loads of gay kids at school, no one cared…I even played the lead female character in the school play and everyone thought it was great. Disclosing HIV status was trickier – it definitely felt like something to be ashamed of as it wasn’t something that was ever talked about with friends when growing up. Like, the HIV/AIDS thing was before our time” (33 years old, 10 years since diagnosis).

For this participant, the generational differences in acceptance toward varying sexual orientations enabled him to feel comfortable coming out during high school. However, the lack of HIV awareness among young gay men during the early 2000s contributed to an internalized stigma toward his chronic health condition.

### Factors That Strengthen Resilience Development

**Fig. 1:**
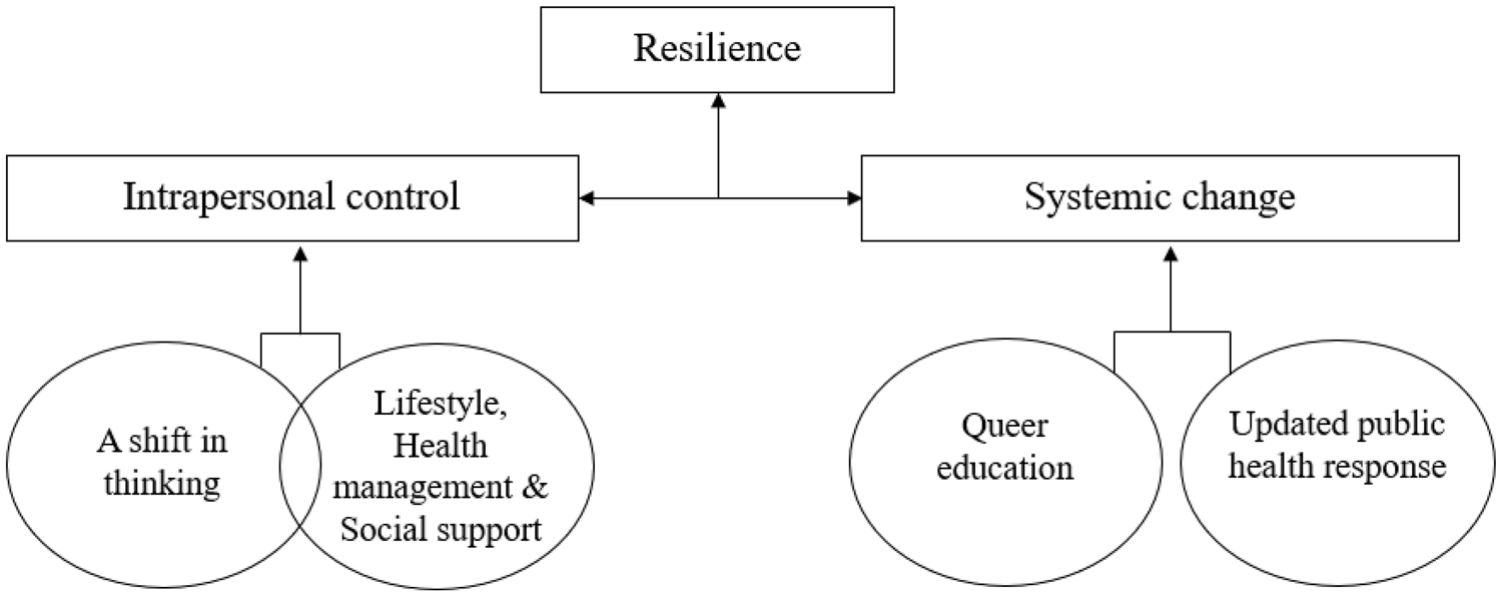
Illustration of primary themes and subthemes generated from the reflexive thematic analysis

Men in this sample expressed ways in which they developed resilience in response to both sexual orientation and HIV-related stigmas. Notably, two primary themes were generated from the data with each theme containing two subthemes. The first theme, “intrapersonal control,” encompasses both cognitive and practical ways in which participants reconciled their sexual orientation and HIV statuses and reduced the impact of these two stigmas on mental health which resulted in an increased sense of resilience. The two sub-themes identified within intrapersonal control included (1) a shift in thinking and (2) lifestyle, health management, and social support. The two subthemes partially intersect and influence each other (see Fig. 1).

The second theme identified, “systemic change,” encompassed how HIV public awareness and messaging impacted the men’s resilience development. This theme included two subthemes (1) queer education and (2) updated public health responses. The subthemes in “systemic change” did not intersect. Although similarities in messaging were identified, target audiences remained nuanced. For example, prevention and treatment information aimed at gay men (regardless of HIV status) is needed to combat in-group stigma, whereas public awareness of HIV transmission and prevention tackles the same issue (e.g., HIV treatment and prevention understanding) but is aimed at an audience wider than gay men. Themes are displayed in Fig. 1 and discussed in depth below.

### Intrapersonal Control

This theme relates to how participants utilize their internal ability to manage negative thoughts or emotions related to their HIV diagnosis or sexual orientation. Participants expressed how taking control of their mindset as well as implementing the practical steps required for successful health management (e.g., medication adherence) were crucial for developing resilience toward internalized stigmas. Participants achieved this by accessing up-to-date HIV information and education, which increased their motivation to accept and respond proactively to their diagnosis, rather than avoid it. This encouraged participants to reappraise their health condition and reduce negative thinking. For example:

After I was diagnosed, I thought I was going to have to take 20 pills each day to stay alive. I had already decided that my life was over… I read up on meds and side effects and realized that it’s like two pills per day. Easy. Just reading that made me sleep better (40 years old, eight years since diagnosis).

Similarly, learning about how combination antiretroviral therapy can lead to an undetectable viral load helped participants focus on an attainable goal of viral suppression, which strengthened their resilience by reaffirming that HIV is a manageable health condition. For example, one participant stated:

Google is your friend. Don’t be scared to research medications. [HIV treatment] information is easy to find on the internet and you can learn how easy it is to manage [HIV] now. There are loads of links about being undetectable, forums on what to ask your doctor about medications et cetera (58 years old, 15 years since diagnosis).

When participants felt in control of their diagnosis, which was enhanced by proactively learning about their condition, they reported a shift in their thinking and motivation to change lifestyle behaviors.

#### A Shift in Thinking

Participants identified three factors: increased self-awareness, reappraisal, and self-efficacy, which they believed led to a shift in their thinking toward their stigmatized identities and subsequently produced changes in both their attitudes and behavior. Although the relationships among the three factors were not linear, the factors interacted with each other. For example, participants expressed that once they had recognized adverse behaviors (e.g., problematic negative thought patterns), they were better able to challenge and change the way they approached adverse situations having and the self-belief to make such changes. For example, through individual counseling, one participant reflected that he recognized “HIV is just one part of my life and doesn’t define me. I am more than HIV” (33 years old, 10 years since diagnosis). Through accessing regular counseling sessions and learning how to reappraise HIV from a disastrous to a manageable chronic health condition, this participant was able to challenge and change his automatic negative beliefs about his HIV diagnosis. Moreover, participants who expressed self-awareness related to unhelpful negative thinking patterns were better able to foster self-efficacy toward personal HIV health management. Participants expressed when they confronted their diagnosis with a problem-solving mindset and shifted focus away from feelings of fault, they felt compelled to manage their health condition. For example, one participant stated “I have two choices. I can hate and pity myself or I can learn about this [condition] and get on with it” (39 years old, eight years since diagnosis). Likewise, participants expressed that reappraising HIV from a moral or social judgment to a medically manageable virus was crucial in strengthening resilience. For example:

As soon as I acknowledged that I did not have to feel guilty about contracting a virus that does not discriminate, I could start to move forward with my life. I don’t feel shame now. I just had sex. That was it. I didn’t ask for this virus (52 years old, 26 years since diagnosis).

Participants expressed that when they approached their HIV diagnosis with a proactivity to understand their health condition and acknowledged that living with HIV was no longer a death sentence or something that defined them, an increased sense of control and motivation to manage their health condition was achieved. Further, acceptance of HIV status impacted on status disclosure and expressed similarly to sexual orientation disclosure, for example, “having HIV is like coming out twice. It’s terrifying. When you’ve done it once though, it gets easier” (52 years old, 12 years since diagnosis). Participants in this sample paralleled their experiences of sexual orientation and HIV status disclosures and expressed how both require internal reappraisal to accept and respond to stigma adaptively. One participant stated, “I’m no longer the ‘AIDS-ridden faggot’ that my brother once called me…I’m now back to being me” (53 years old, 14 years since diagnosis), demonstrating a shift in thinking toward dual stigmatized identities is possible, and reclaiming one’s identity through reappraisal enhances personal resilience.

#### Lifestyle, Health Management, and Social Support

Influenced by a shift in thinking, participants expressed that practical lifestyle changes and HIV management further enhanced their sense of control toward their HIV status. Participants articulated how greater intrapersonal control aligned with increased adaptive lifestyle behaviors. For example, “I can’t know or control everything [about HIV], but what I can, like diet, exercise, blood work, meds etc., why wouldn’t I? I can’t un-do it [HIV] but I can keep it undetectable and not let it rule my life” (43 years old, six years since diagnosis).

All participants in this study attended regular medical appointments, adhered to medication regimes, and were virally undetectable. Over three-quarters of the participants described how they felt motivated to engage in adaptive lifestyle changes as part of their HIV management strategies. For instance, “I’ve reduced how much I drink during the week and join in with exercise classes [at the HIV center]” (59 years old, seven years since diagnosis). Participants who were not as keen to sustain lifestyle changes, such as regular exercise (25%), attributed this to comorbid health conditions such as arthritis or age-related immobility issues. Nonetheless, all participants reported a history of positive change toward diet and physical activity within 12–18 months of initial HIV diagnosis and attributed these changes to a problem-solving approach to managing their health.

The men who continued to engage in weekly recreational alcohol and/or drug use (33%) reported trying new ways of managing stress such as using mindfulness or meditation techniques, for example, “after 12 months of spiraling behavior [drugs and unprotected sex] I decided I had to make some changes. Meditation, mindful walking-groups, drug free days etc.” (43 years old, six years since diagnosis). Although alcohol and substance use were ascribed as coping mechanisms, men who were frequent users reported a reduction in use since HIV diagnosis. This was motivated by a desire to avoid negative interaction effects between alcohol/substances and HIV-medication and the impact on their immune system.

As well as implementing practical lifestyle changes as part of their health management strategies, the men in this study shared that accessing social support was equally as important in fostering resilience. All participants had experienced a form of social support in relation to sexual orientation status or HIV diagnosis (e.g., friends, family, or peer support). In particular, participants expressed that having access to other gay men with HIV helped them ask questions without embarrassment or feelings of shame. For example:

I was only out of the closet for six months before I was diagnosed with HIV. It felt like a double whammy. My family were just getting their heads around me coming out [as gay]. To then tell them I have HIV … well, I wanted to hide. Luckily, I have great support here [at the HIV service] which helped me process being gay, and my HIV diagnosis without judgment (59 years old, seven years since diagnosis).

Further, access to an HIV center and group peer-support helped men navigate concerns regarding HIV status and intimate relationships post-diagnosis. One participant commented:

My family are fantastic support but don’t always get what it’s like… dating with HIV can be scary. A few guys shared their experiences of how they disclosed status to sexual partners…it’s taken the edge off having these conversations now I feel like I can own it [HIV] and not be shamed by it*.* (33 years old, 10 years since diagnosis).

Similarly, men who accessed support related to sexual orientation concerns (e.g., via counseling, gay support groups or from LGBTQIA+ allies) expressed how important the support was for developing a strong sense of self related to sexual identity. For example, “I grew up in the country where it’s still considered a problem if you’re gay. I got on to some of the phone support groups…they helped me process and understand who I am…I wouldn’t be here today if didn’t do that” (58 years old, 15 years since diagnosis). This participant expressed how constant exposure to homophobia in his country town caused suicidal ideation. However, through support that provided acceptance strategies together with help in acknowledging and owning his sexual orientation, he was able to express his full self.

### Systemic Change

A second primary theme, “systemic change,” emphasized the importance of external messaging related to HIV treatment and current messaging regarding HIV transmission in the wider community (e.g., HIV notifications among gay versus heterosexual sexual orientations in Australia) and how a lack of such messaging has impeded the development of resilience for those living with the virus. For example, participants felt there was a lack of current information outside of medical settings or HIV services with one man stating “we hear about cancer treatments all the time…how they advance…where’s the [public] information about HIV treatment and reduced transmission [in the gay community?]” (62 years old, 31 years since diagnosis). Similarly, participants felt forgotten by health campaigns which focussed on HIV prevention and not treatment, with one man commenting “the younger generation know all about PrEP but what do they know about viral suppression?” (43 years old, 10 years since diagnosis). Additionally, another participant stated, “it makes me feel like shit that HIV is still seen as a gay man’s disease…like we’re the outcasts” (58 years old, 15 years since diagnosis). As such, participants expressed that a lack of updated public messaging about biomedicine, viral suppression, and transmission risk continued to contribute to societal stigma about HIV and decreased the resilience among those living with the virus.

#### Queer Education

Men in this study also voiced concerns related to a lack of understanding by others about HIV treatment efficacy, specifically among HIV-negative gay men. Some of the men expressed how this lack of awareness can impact on attitudes toward having sex with HIV-positive men, which in turn inhibited their personal resilience toward HIV stigma. For example:

I get asked if I’m “clean” all the time. What does that even mean? I’m undetectable. I have regular blood tests…do you? There needs to be a more concerted effort to stamp out this kind of language … and educate people on what viral suppression means (39 years old, eight years since diagnosis).

Participants suggested that current messaging toward gay men communicating the effectiveness of ART and U = U could be stronger, similar to how information about preventative medications such as pre-exposure prophylaxis (PrEP) is currently communicated in the gay community. One participant reflected:

I was called a dirty bastard when I disclosed my undetectable HIV status [to a recent sexual partner]. This guy was on PrEP! He didn’t understand I am undetectable – I also had condoms. He just saw me as someone disgusting with AIDS (43 years old, six years since diagnosis).

Several participants sensed a divide between those with HIV and HIV-negative gay men who take preventative medication. These men explained this as a type of hierarchy (e.g., “it’s like them versus us…we’re looked down upon” [62 years old, 31 years since diagnosis]), where virally suppressed HIV-positive men are assumed to be a transmission risk. Similarly, one participant commented that after a sexual encounter, where HIV status and viral suppression had been disclosed, he received a text message asking, “I want it in writing that you’re taking meds…the month you started, or I’ll take legal action” (39 years old, eight years since diagnosis). This participant stated that the sexual partner was on PrEP yet stigmatized him for his HIV status. This resulted in anger, frustration, and a lack of confidence toward social media-app dating. As such, stigma within the gay community was expressed as problematic and associated with the systemic issue of societal stigma exacerbated by a lack of effective messaging within the gay community.

#### Updated Public Health Responses

Approximately three-quarters of the participants reported they had moved to Melbourne city or inner-city suburbs from regional areas due to greater availability of HIV services and LGBTQIA+ inclusive health-care options. Men who reported negative health care experiences from regional health-care providers related to sexual orientation or HIV status expressed increased feelings of shame, guilt, and fear of ostracization from their local communities. For example, one participant reported his experience of accessing medical treatment when visiting his home town, commenting “the nurse was doubled-gloved and wore a face shield like I had Ebola or something just as contagious” (62 years old, 31 years since diagnosis). Participants who had moved from regional areas agreed that better public health information regarding sexual health management and access to HIV services is lacking outside of Australian metropolitan areas.

Further, most men in this study voiced disappointment with the lack of current mainstream messaging regarding HIV transmission statistics in Australia, with one commenting “you know it’s not just us anymore…heaps of straights get it [HIV] as well nowadays but they don’t promote that” (67 years old, 36 years since diagnosis). Similarly, frustration was expressed by another participant with regard to the lack of public awareness campaigns promoting advancements in biomedicine, for example, “where’s the updated adverts? Let’s tell the wider community about PrEP, ART and viral suppression” (59 years of age, 19 years since diagnosis). Without updated messaging, men felt that community awareness of HIV was still very much associated with sexual orientation, impacting on resilience toward both sexual orientation and HIV-related stigmas. One participant expressed how an historic “scare-tactic” television advertisement from 1980’s and 1990’s was experienced:

Do you remember that awful AIDS Grim Reaper TV ad from the ‘90 s? Horrifying. It scared me shitless. There was no way I was going to come out to my friends and family as gay in my home town after seeing that…How on earth would they accept my HIV diagnosis years later? (51 years of age, 24 years since diagnosis).

Participants agreed that the lack of updated public health campaigns contributed toward the systemic issues of sexual orientation and HIV-related stigmas. They felt that better public health messaging would help influence attitudes toward HIV from the general population.

## Discussion

The aim of this qualitative study was to examine how Australian gay men strengthened their resilience toward sexual orientation and HIV-related stigmas. The data we obtained showed that the men in this study expressed how both sexual orientation and HIV-related stigmas have intertwined to negatively impact on their mental health outcomes over the years. Despite this, however, the men have responded in adaptive and resilient ways to their dual stigmatized identities. We found that their resilience was strengthened through intrapersonal and social support and through systemic means. The present findings build on the extant literature which demonstrates that Australian gay men can increase their resilience toward stigmatized identities (Lyons & Heywood, 2016; Lyons et al., 2016) and suggests ways resilience can be enhanced. The interviews elucidated the ways in which this sample of HIV-positive gay men achieved greater resilience. Importantly, the findings in this study support the need for a multifaceted approach toward examination of stigma experience (e.g., consideration of intersecting stigmatized identities) and how co-occurring stigmas impact on resilience development (e.g., interpersonal, intrapersonal, and availability of community/social support) (Dulin et al., 2018; Earnshaw et al., 2022). Further, the men in this study experienced sexual orientation and HIV stigmas in similar ways (e.g., rejection sensitivity and heightened concealment motivation), aligning to findings from previous minority subgroup research (Meyer, 1995, 2003; Rendina et al., 2017).

This discussion considers the implications of these findings from how we conceptualize resilience in gay men and also considers the implications for social policy.

### Sources of Resilience

#### Intrapersonal Control

Participants developed resilience at an individual level through self-awareness (e.g., recognition of negative thinking), reappraisal of negative thinking patterns (e.g., HIV does not define me; being gay is okay), and self-efficacy (e.g., belief in self to make adaptive lifestyle changes and sustain viral suppression). Men’s intrapersonal control was further strengthened when they engaged social support (e.g., peer support and access to LGBTQIA+ and HIV-related health services). Moreover, men in this study expressed how access to LGBTQIA+ inclusive service providers is important for men struggling with their sexual orientation, as well as access to HIV-related services outside of metropolitan areas. Such services were considered as essential in helping men navigate challenging adverse life events related both sexual orientation and HIV management decisions.

Importantly, 90% participants in this study were over 40 years of age suggesting life experience might strengthen resilience toward adverse life events compared to younger adults with less lived experience (Fumaz et al., 2015). For example, individuals who have experienced more life adversities can draw on both individual and environmental resources, might better be able to negotiate, adapt to, or manage significant sources of stress or trauma (Windle, 2011). Participants in this study who grew up during eras where homosexuality was not normalized, and those who lived through the HIV/AIDS epidemic in Australia have likely developed such management skills toward dual stigmatized identities. Moreover, all participants in this study have had access to both individual and environmental resources (e.g., counseling, peer support, and community/connectedness), which has helped them strengthen their responses to stressful or traumatic stigma experiences.

Although individuals in this sample have learned to respond adaptively to their stigmatized identities, the lasting impact of stigma continues to negatively affect their mental health, potentially in different ways between age groups. For example, sexual orientation disclosure was less problematic for a younger participant who grew up during the early 2000s, compared to the older participants who matured during times where homosexuality was illegal or heavily stigmatized by society and in the media, for example, increased violence and hate crimes. The impact of this has resulted in lasting trauma for many individuals, negatively impacting on mental health. Similarly, HIV stigma was perceived differently among men who experienced the early years of the pandemic, compared to younger men who grew up without the experience of witnessing AIDS related deaths, or experience of blame and HIV-related community hysteria. As such, the heterogeneity of stigma experience and resilience development needs to be considered in future resilience-based workshops (Fredriksen-Goldsen et al., 2017), which could be adapted for age of participants and duration of HIV-diagnosis.

Resilience development was also fostered through access to others’ lived experience (e.g., through HIV centers and support groups), which helped men re-assess their own internal responses to both internalized and external stigmas and provided suggested coping strategies (e.g., mindfulness, advice on sexual orientation or HIV disclosure, and dating with HIV). The current findings reflect Canadian studies (Liboro et al., 2021a,b) that found access to HIV information and support services fostered internal and external resilience and are important factors in HIV health management. Sadly, access to such services is not fully equitable throughout Australia and participants in this study moved to the city for such services. Moreover, like previous research on the impact of collective resilience (Lyons & Heywood, 2016), men in this study expressed greater self-efficacy and implementation of emotional control and behavioral change when hearing about how other HIV-positive men have successfully managed sexual orientation and HIV stigmas.

Men in this study drew on situational reappraisal and behavioral change strategies, which draw similar comparisons to clinical interventions that include cognitive restructuring and emotion regulation strategies. For instance, research has shown that individuals who reappraise catastrophic thinking (e.g., around efficacy of treatment outcomes and life expectancy post diagnosis) are better able to ameliorate negative self-concept, increase psychological wellbeing, and regulate emotion responses toward stigma (Chan & Mak, 2019; Gross, 1998; Rendina et al., 2017). Further, the current participants showed a shift in thinking toward their HIV status not only when they re-appraised long-term health outcomes (e.g., the efficacy medication adherence on viral suppression) but also when they heard from resilient men who have experienced both sexual orientation and HIV stigmas. This speaks to importance of access to peer-led support and the generativity of lived experience fostering wellbeing among newly diagnosed individuals (Bower et al., 2021).

#### Systemic Change

Strengthening of resilience should not be confined to simply reappraisal of negative thinking or adaptive responses to negative interpersonal experiences (Pantelic et al., 2019). In the current study, participants identified how external social and structural processes impact on individual stigma experience and resilience development. Men in this study identified a need for better HIV treatment education within the gay community (e.g., ART and viral suppression information), as well as greater public health messaging related to HIV transmission within the wider community (e.g., heterosexual transmission notifications). Men expressed that they felt HIV messaging was primarily focussed within the gay community and a lack of visible government awareness campaigns in the wider community promoted a stigmatized and outdated view of HIV transmission in Australia.

This is disappointing, as in the last five-years Australia, has seen a 45% decrease of male-to-male HIV-infection notifications (Kirby Institute, 2020). However, men in this study felt the lack of balanced messaging related to these statistics has inhibited their resilience development. This was interpreted as an issue that is out of their control (e.g., individual resource), but could be essential in helping them feel less ostracized if public messaging outlining current HIV information was available (e.g., community-level resources). As such, it is important to address resilience development through multiple sources and not simply focus on an individual’s ability to cope with or reappraise situations that negatively impact on physical and mental health (Gabbidon et al., 2022).

Interestingly, men in this study also expressed experiences of in-group HIV stigma from HIV-negative men within the gay community. This contrasts with accounts of “gay camaraderie” during the peak of the HIV/AIDS crisis (McKinnon et al., 2017), whereby the gay community rallied together to support those who were dying of the virus. Although research suggests a “serostatus divide” among gay men could be related to HIV risk reduction within sexual relationships (de Wit et al., 2013), it is evident from the present interview data that the ostracization experienced by the gay men in this study had negatively impacted their capacity to develop resilience and a sense of self-worth. Future research could examine the current concept of “gay community” among younger cohorts of gay men, to understand if there is a desire to support all gay men regardless of their HIV status, and if this does not exist, what might help enable it. This could help researchers to understand how young gay men experience sexual orientation and HIV stigma since the introduction and uptake of PrEP and if a hierarchy exists between those living with HIV versus those taking preventative medication. This would help inform researchers of current stigma sources within the gay community and adapt resilience interventions accordingly.

### Social Policy Implications

The findings from this study inform recommendations and have implications for social policy. Australians benefit from access to universal healthcare and free or low-cost medications supported by the pharmaceutical benefit scheme (PBS). Further, individuals who meet criteria for disability support can also access benefits for a range of allied health services through the National Disability Insurance Scheme (NDIS). However, even with such services available, many participants in this study identified gaps and obstacles that negatively affected their wellbeing. For example, access to LGBTQIA+ inclusive medical providers are still primarily limited to metropolitan areas or capital cities. Such limitations create barriers to accessing appropriate services (e.g., gay men’s health literacy services) and heighten perceived stigma and discrimination if seeking testing, ART or PrEP (Brona Nic Giolla et al., 2022; Lea et al., 2019). Similarly, access to HIV support services outside of urban areas is limited yet crucial in providing support and information for HIV-positive individuals. As such, policy makers should aim to support such initiatives for greater regional accessibility, for face-to-face services or increased telehealth support, the latter of which has been demonstrated to be successful during COVID-19 lockdowns in Australia (Reay et al., 2021).

Men in this study also referred to the lack of current HIV treatment and transmission awareness campaigns throughout Australia. Outside of LGBTQIA+ venues and universities, minimal national advertising is seen in relation to the importance of viral suppression (e.g., U = U), PrEP, or safe-sex awareness. These types of campaigns would educate a broader audience and enhance sexual health literacy and might also help to reduce the historical damaging narrative of a gay sexual orientation being synonymous with HIV/AIDS infection (Fela, 2019). Helping dismantle damaging negative stereotypes of gay men and HIV status, a parliamentary inquiry is being held (at time of writing) to investigate LGBTQIA+ hate crimes between 1970 and 2010. Part of this inquiry is examining how advertisements such as the government’s Grim Reaper AIDS campaign from the 1980s contributed to overt abuse, heightened tensions in the gay community, and provided justification for homophobic violence (McKinnell, 2022). Since the Grim Reaper advertisement aired, there have been local campaigns highlighting the efficacy of treatment as prevention (TasP) (e.g., U = U) advertised within the LGBTQIA+ community; however, no national government campaigns have taken place. Although participants from the current study suggested a lack of national awareness campaigns have contributed to stigma experiences and inhibited resilience development, messaging that does not encourage HIV-stigma or a sense of “othering” between those with HIV and the wider community needs to be addressed.

The National Association of People with HIV Australia (NAPWHA, 2022) explored attitudes and beliefs toward individuals with HIV. Their unpublished report, which included predominantly suburban or regional heterosexual males and families with strongly held conservative cultural beliefs, assessed the effectiveness of HIV messaging outside of the LGBTQIA+ community. Their findings indicated national campaigns related to HIV transmission and messaging regarding the effectiveness of TasP are unlikely to succeed due to the “self-distancing” that occurs between those without the virus and those living with HIV. For example, attitudes toward people living with HIV (PLHIV) included blame regarding contracting the virus (e.g., promiscuity and participation in gay sex) and participants perceived PLHIV as irresponsible and a threat toward community safety. Although TasP was seen as a positive treatment option, doubt regarding the efficacy of such treatment was high due to lack of awareness and understanding of viral suppression. As such, before national campaigns or awareness messaging is revised that promotes TasP to a wider audience, campaigns, or interventions that help challenge the negative fixed beliefs about PLHIV are first needed.

### Strengths, Limitations, and Future Directions

This qualitative study adds to the small but growing body of Australian-based research, which identifies strategies to increase resilience among HIV-positive gay men. The current findings can be used to assist healthcare providers to enhance resilience-based programs, with a focus on intrapersonal factors such as developing self-awareness, reappraisal techniques, and self-efficacy tools. However, the research is not without limitations. Importantly, the sample lacked racial diversity (all participants identified as white/Anglo/European). As such, it is unclear if resilience is strengthened in the same way by gay men who face greater intersectional-stigma (e.g., First Nations People and culturally and linguistically diverse Australians). Melbourne is considered a multicultural city (Nelson & Ho, 2020), and recruitment was conducted in an inner-city suburb. However, barriers to health-seeking behavior such as community-ostracism, health literacy, or religious factors (e.g., taboos) (Ziersch et al., 2021) could account for the lack of cultural representation. As such, multiple stigmatized identities (e.g., intersectional stigma experience) and the impact on resilience development should be considered in future Australian research to better inform treatment outcomes, which could vary between cultures or background.

The current sample also consisted mainly of older middle-aged adults and older-adults to retirement age who were under medical management. Participants were all virally suppressed with and average time since diagnosis of over 16 years. It might be considered that this cohort of participants, with extensive life and HIV management experience, is simply coping well toward dual stigmatized identities, due to having to cope during times when being gay or having HIV was considered differently. Future research would benefit from examining younger cohorts of gay men at different stages of HIV treatment to explore their experience of stigma and measure resilience responses to both sexual orientation and HIV-related stigma. A younger cohort of gay men who have not experienced illegality of sexual orientation, same-sex marriage, or an HIV epidemic is likely to have different perspectives and lived experiences of how both sexual orientation and HIV-related stigmas interact.

Finally, all men in this sample were regular participants of an inner-city HIV service and lived in the local area. They do not reflect perspectives of men in regional communities who do not have equitable access to LGBTQIA+ and HIV-related services. Research examining adjustment challenges of men in regional and remote Australia will provide further insights on coping strategies and resilience-development that can be applied to rural treatment interventions or resilience-based programs.

## Conclusion

Findings from this study highlight that HIV-positive gay men in Australia experience a variety of stigmas related to their dual stigmatized identities. Despite this, the men demonstrated that resilience toward both sexual orientation and HIV-related stigmas can be strengthened through intrapersonal control (e.g., self-awareness, reappraisal, and self-efficacy related to their chronic health condition and treatment outcomes) and systemic changes (such as updated HIV awareness campaigns and messaging that aims to humanize PLHIV). Findings from this study also demonstrate that policy makers in Australia would benefit from working with HIV organizations and advocates to promote HIV treatment and prevention in a non-stigmatizing way that is directed toward the boarder community and is focused on creating allies. Finally, continued funding to support LGBTQIA+ inclusive healthcare providers as well promoting greater accessibility of HIV-related services is required throughout Australia, especially within regional and remote areas, to ensure individuals are not simply defined by their sexual orientation or HIV status.

## Data Availability

Data is available on request due to privacy and ethical restrictions. The data that support the findings of this study are available on request from the corresponding author, NAL. The data are not publicly available due to containing information that could compromise the privacy of research participants.

## Code Availability

N/A.

## References

[CR1] Algarin AB, Sheehan DM, Varas-Diaz N, Fennie KP, Zhou Z, Spencer EC, Cook RL, Morano JP, Ibanez GE (2020). Health care-specific enacted HIV-related stigma's association with antiretroviral therapy adherence and viral suppression among people living with HIV in Florida. AIDS Patient Care and STDs.

[CR2] Arístegui I, Radusky PD, Zalazar V, Lucas M, Sued O (2018). Resources to cope with stigma related to HIV satus, gender identity, and sexual orientation in gay men and transgender women. Journal of Health Psychology.

[CR3] Asakura K, Craig SL (2014). “It gets better”… but how? Exploring resilience development in the accounts of LGBTQ adults. Journal of Human Behavior in the Social Environment.

[CR4] Australian Bureau of Statistics. (2014). *Age standard*. version-1.7. ABS. https://www.abs.gov.au/statistics/standards/age-standard/latest-release

[CR5] Ayhan CHB, Bilgin H, Uluman OT, Sukut O, Yilmaz S, Buzlu S (2020). A systematic review of the discrimination against sexual and gender minority in health care settings. International Journal of Health Services.

[CR6] Blanch J, Rousaud A, Martinez E, De Lazzari E, Peri J-M, Milinkovic A, Perez-Cuevas J-B, Blanco J-L, Gatell J-M (2002). Impact of lipodystrophy on the quality of life of HIV-1-infected patients. Journal of Acquired Immune Deficiency Syndromes.

[CR7] Bower KL, Lewis DC, Bermúdez JM, Singh AA (2021). Narratives of generativity and resilience among LGBT older adults: Leaving positive legacies despite social stigma and collective trauma. Journal of Homosexuality.

[CR8] Bränström R, Pachankis JE (2019). The role of country-level structural stigma on sexual orientation disclosure and discrimination in health care settings among lesbian, gay, and bisexuals across Europe. European Journal of Public Health.

[CR9] Braun V, Clarke V (2020). One size fits all? What counts as quality practice in (reflexive) thematic analysis? Qualitative research in psychology. Advance Online Publication.

[CR10] Brewer R, Hood KB, Moore M, Spieldenner A, Daunis C, Mukherjee S, Smith-Davis M, Brown G, Bowen B, Schneider JA (2020). An exploratory study of resilience, HIV-related stigma, and HIV care outcomes among men who have sex with men (msm) living with HIV in Louisiana. AIDS and Behavior.

[CR11] Brona Nic Giolla, E., Reynish, T. D., Ha, H., Bridgman, H., Corvinus-Jones, S. L., & Auckland, S. (2022). A systematic review of the health and health care of rural sexual and gender minorities in the UK, USA, Canada, Australia and New Zealand. *Rural Remote Health, 22*(3), 6999–6999. 10.22605/RRH6999 10.22605/RRH6999 35794784

[CR12] Brown G, Leonard W, Lyons A, Power J, Sander D, McColl W, Johnson R, James C, Hodson M, Carman M (2017). Stigma, gay men and biomedical prevention: The challenges and opportunities of a rapidly changing HIV prevention landscape. Sexual Health.

[CR13] Cama E, Brener L, Slavin S, de Wit J (2020). The relationship between negative responses to HIV status disclosure and psychosocial outcomes among people living with HIV. Journal of Health Psychology.

[CR14] Cambiano, V., Bruun, T., Degen, O., Geretti, A. M., Raben, D., Coll, P., Antinori, A., Weber, R., Van Eeden, A., Raffi, F., Wandeler, G., Gerstoft, J., Kitchen, M., Leon, A., Ristola, M., Lundgren, J., Coll, P., Meulbroek, M., Carrillo, A., ... Gussio, M. (2019). Risk of HIV transmission through condomless sex in serodifferent gay couples with the HIV-positive partner taking suppressive antiretroviral therapy (partner): Final results of a multicentre, prospective, observational study. *The Lancet (British edition), 393*(10189), 2428–2438. 10.1016/S0140-6736(19)30418-0 10.1016/S0140-6736(19)30418-0 PMC6584382 31056293

[CR15] Casey, L. J., Wootton, B. M., & McAloon, J. (2020). Mental health, minority stress, and the Australian marriage law postal survey: A longitudinal study. *American Journal of Orthopsychiatry, 90*(5), 546. 10.1037/ort0000455 10.1037/ort0000455 32338941

[CR16] Chan, R. C. H., & Mak, W. W. S. (2019). Cognitive, regulatory, and interpersonal mechanisms of HIV stigma on the mental and social health of men who have sex with men living with HIV. *American Journal of Men's Health, 13*(5), Article 1557988319873778. 10.1177/1557988319873778 10.1177/1557988319873778 PMC6728686 31690214

[CR17] Cole ER (2009). Intersectionality and research in psychology. American Psychologist.

[CR18] Cronin TJ, Pepping CA, Halford WK, Lyons A (2021). Mental health help-seeking and barriers to service access among lesbian, gay, and bisexual Australians. Australian Psychologist.

[CR19] De Santis JP, Florom-Smith A, Vermeesch A, Barroso S, DeLeon DA (2013). Motivation, management, and mastery: A theory of resilience in the context of HIV infection. Journal of the American Psychiatric Nurses Association.

[CR20] de Wit, J. B. F., Murphy, D. A., Adam, P. C. G., & Donohoe, S. (2013). Strange bedfellows: HIV-related stigma among gay men in Australia. In Liamputtong, P. (Eds.), *Stigma, Discrimination and Living with HIV/AIDS*. (pp. 289–308). Springer Netherlands. 10.1007/978-94-007-6324-1_17

[CR21] Deeks SG, Lewin SR, Havlir DV (2013). The end of AIDS: HIV infection as a chronic disease. The Lancet.

[CR22] Dulin, A. J., Dale, S. K., Earnshaw, V. A., Fava, J. L., Mugavero, M. J., Napravnik, S., Hogan, J. W., Carey, M. P., & Howe, C. J. (2018, 2018/08/23). Resilience and HIV: A review of the definition and study of resilience. *AIDS Care, 30*(sup5), S6-S17. 10.1080/09540121.2018.1515470 10.1080/09540121.2018.1515470 PMC6436992 30632778

[CR23] Earnshaw VA, Jonathon Rendina H, Bauer GR, Bonett S, Bowleg L, Carter J, English D, Friedman MR, Hatzenbuehler ML, Johnson MO (2022). Methods in HIV-related intersectional stigma research: Core elements and opportunities. American Journal of Public Health.

[CR24] Ecker S, Riggle ED, Rostosky SS, Byrnes JM (2019). Impact of the Australian marriage equality postal survey and debate on psychological distress among lesbian, gay, bisexual, transgender, intersex and queer/questioning people and allies. Australian Journal of Psychology.

[CR25] Eisinger RW, Dieffenbach CW, Fauci AS (2019). HIV viral load and transmissibility of HIV infection: Undetectable equals untransmittable. JAMA.

[CR26] Emlet CA, Tozay S, Raveis VH (2011). “I’m not going to die from the AIDS”: Resilience in aging with HIV disease. The Gerontologist.

[CR27] Fela, G. (2019). 'A shudder of terror': HIV/AIDS nursing, oral history and the politics of emotion. *Oral History Australia Journal, 41*, 50–56. 10.3316/informit.912536838750012

[CR28] Fingerhut AW, Peplau LA, Gable SL (2010). Identity, minority stress and psychological well-being among gay men and lesbians. Psychology and Sexuality.

[CR29] Fredriksen-Goldsen KI, Shiu C, Bryan AE, Goldsen J, Kim H-J (2017). Health equity and aging of bisexual older adults: Pathways of risk and resilience. The Journals of Gerontology: Series B.

[CR30] Fumaz CR, Ayestaran A, Perez-Alvarez N, Muñoz-Moreno JA, Moltó J, Ferrer MJ, Clotet B (2015). Resilience, ageing, and quality of life in long-term diagnosed HIV-infected patients. AIDS Care.

[CR31] Gabbidon K, Chenneville T, Earnshaw V, Drake H (2022). Intersectional stigma and developmental competence among youth living with HIV. Journal of Family Theory & Review.

[CR32] Goldenberg T, Stephenson R, Bauermeister J (2018). Community stigma, internalized homonegativity, enacted stigma, and HIV testing among young men who have sex with men. Journal of Community Psychology.

[CR33] Gottert A, Friedland B, Geibel S, Nyblade L, Baral SD, Kentutsi S, Mallouris C, Sprague L, Hows J, Anam F, Amanyeiwe U, Pulerwitz J (2019). The people living with HIV (PLHIV) resilience scale: Development and validation in three countries in the context of the PLHIV stigma index. AIDS and Behavior.

[CR34] Gross JJ (1998). The emerging field of emotion regulation: An integrative review. Review of General Psychology.

[CR35] Harper GW, Bruce D, Hosek SG, Fernandez MI, Rood BA (2014). Resilience processes demonstrated by young gay and bisexual men living with HIV: Implications for intervention. AIDS Patient Care and STDs.

[CR36] Hatzenbuehler ML, Pachankis JE (2016). Stigma and minority stress as social determinants of health among lesbian, gay, bisexual, and transgender youth: Research evidence and clinical implications. Pediatric Clinics of North America.

[CR37] Herek GM, Gillis JR, Cogan JC (2015). Internalized stigma among sexual minority adults: Insights from a social psychological perspective. Journal of Counseling Psychology.

[CR38] Hooghe M, Meeusen C (2013). Is same-sex marriage legislation related to attitudes toward homosexuality?. Sexuality Research and Social Policy.

[CR39] Hubach, R. D., Dodge, B., Schick, V., Ramos, W. D., Herbenick, D., Li, M. J., Cola, T., & Reece, M. (2015, 2015/08/09). Experiences of HIV-positive gay, bisexual and other men who have sex with men residing in relatively rural areas. *Culture, Health and Sexuality, 17*(7), 795–809. 10.1080/13691058.2014.994231 10.1080/13691058.2014.994231 25608847

[CR40] Hussen, S. A., Harper, G. W., Rodgers, C. R. R., van den Berg, J. J., Dowshen, N., & Hightow-Weidman, L. B. (2017, Aug). Cognitive and behavioral resilience among young gay and bisexual men living with HIV. *LGBT Health, 4*(4), 275–282. 10.1089/lgbt.2016.0135 10.1089/lgbt.2016.0135 PMC5564056 29792564

[CR41] Iott BE, Loveluck J, Benton A, Golson L, Kahle E, Lam J, Bauermeister JA, Veinot TC (2022). The impact of stigma on HIV testing decisions for gay, bisexual, queer and other men who have sex with men: A qualitative study. BMC Public Health.

[CR42] Kirby Institute. (2020). *HIV, viral hepatitis and sexually transmissible infections in Australia: Annual surveillance report 2020*. https://kirby.unsw.edu.au/sites/default/files/kirby/report/Annual-Suveillance-Report-2021_HIV.pdf

[CR43] Lea T, Anning M, Wagner S, Owen L, Howes F, Holt M (2019). Barriers to accessing HIV and sexual health services among gay men in Tasmania, Australia. Journal of Gay and Lesbian Social Services.

[CR44] Lea T, Wit J, Reynolds R (2014). Minority stress in lesbian, gay, and bisexual young adults in Australia: Associations with psychological distress, suicidality, and substance use. Archives of Sexual Behavior.

[CR45] Lelutiu-Weinberger C, Pachankis JE, Golub SA, Ja’Nina, J. W., Bamonte, A. J., & Parsons, J. T.  (2013). Age cohort differences in the effects of gay-related stigma, anxiety and identification with the gay community on sexual risk and substance use. AIDS and Behavior.

[CR46] Liboro, R., Despres, J., Ranuschio, B., Bell, S., & Barnes, L. (2021a). Forging resilience to HIV/AIDS: Personal strengths of middle-aged and older gay, bisexual, and other men who have sex with men living with HIV/AIDS. *American Journal of Men's Health, 15*(5). 10.1177/15579883211049016 10.1177/15579883211049016 PMC8488414 34587823

[CR47] Liboro R, Yates TC, Bell S, Ranuschio B, Da Silva G, Fehr C, Ibañez-Carrasco F, Shuper PA (2021). Protective factors that foster resilience to HIV/AIDS: Insights and lived experiences of older gay, bisexual, and other men who have sex with men. International Journal of Environmental Research and Public Health.

[CR48] Livingston NA, Flentje A, Brennan J, Mereish EH, Reed O, Cochran BN (2020). Real-time associations between discrimination and anxious and depressed mood among sexual and gender minorities: The moderating effects of lifetime victimization and identity concealment. Psychology of Sexual Orientation and Gender Diversity.

[CR49] Lyons A, Heywood W (2016). Collective resilience as a protective factor for the mental health and well-being of HIV-positive gay men. Psychology of Sexual Orientation and Gender Diversity.

[CR50] Lyons A, Heywood W, Rozbroj T (2016). Psychosocial factors associated with resilience in a national community-based cohort of Australian gay men living with HIV. AIDS and Behavior.

[CR51] Lyons A, Hill AO, McNair R, Carman M, Morris S, Bourne A (2022). Demographic and psychosocial factors associated with recent suicidal ideation and suicide attempts among lesbian, gay, bisexual, pansexual, queer, and asexual (LGBQ) people in Australia: Correlates of suicidality among LGBQ Australians. Journal of Affective Disorders.

[CR52] Lyons A, Hosking W, Rozbroj T (2015). Rural-urban differences in mental health, resilience, stigma, and social support among young Australian gay men. The Journal of Rural Health.

[CR53] Mallory, C., Hasenbush, A., & Sears, B. (2015). *Discrimination and harassment by law enforcement officers in the LGBT community*. https://escholarship.org/uc/item/5663q0w1

[CR54] McKinnell, J. (2022, November 22). Grim Reaper HIV ads 'contributed' to violence against LGBT community, inquiry told. *ABC News.* https://www.abc.net.au/news/

[CR55] McKinnon S, Gorman-Murray A, Dominey-Howes D (2017). Remembering an epidemic during a disaster: Memories of HIV/AIDS, gay male identities and the experience of recent disasters in Australia and New Zealand. Gender, Place and Culture: A Journal of Feminist Geography.

[CR56] Mey A, Plummer D, Dukie S, Rogers GD, O’Sullivan M, Domberelli A (2017). Motivations and barriers to treatment uptake and adherence among people living with HIV in Australia: A mixed-methods systematic review. AIDS and Behavior.

[CR57] Meyer IH (1995). Minority stress and mental health in gay men. Journal of Health and Social Behavior.

[CR58] Meyer IH (2003). Prejudice, social stress, and mental health in lesbian, gay, and bisexual populations: Conceptual issues and research evidence. Psychological Bulletin.

[CR59] Meyer IH (2016). The elusive promise of LGBT equality. American Journal of Public Health.

[CR60] Morandini JS, Blaszczynski A, Dar-Nimrod I, Ross MW (2015). Minority stress and community connectedness among gay, lesbian and bisexual Australians: A comparison of rural and metropolitan localities. Australian and New Zealand Journal of Public Health.

[CR61] NAPWHA (2022). HIV social impact campaign [unpublished report].

[CR62] Nelson, J., & Ho, C. (2020). Multicultural cities. In D. Rogers, A. Keane, T. Alizadeh, & J. Nelson (Eds.), Understanding urbanism (pp. 135–149). Springer Singapore. 10.1007/978-981-15-4386-9_9

[CR63] Pachankis JE (2014). Uncovering clinical principles and techniques to address minority stress, mental health, and related health risks among gay and bisexual men. Clinical Psychology: Science and Practice.

[CR64] Pachankis JE, Mahon CP, Jackson SD, Fetzner BK, Bränström R (2020). Sexual orientation concealment and mental health: A conceptual and meta-analytic review. Psychological Bulletin.

[CR65] Pantelic M, Sprague L, Stangl AL (2019). It’s not “all in your head”: Critical knowledge gaps on internalized HIV stigma and a call for integrating social and structural conceptualizations. BMC Infectious Diseases.

[CR66] Peleg A, Hartman T (2019). Minority stress in an improved social environment: Lesbian mothers and the burden of proof. Journal of GLBT Family Studies.

[CR67] Power J, Amir S, Lea T, Brown G, Lyons A, Carman M, Rule J, Bourne A (2021). Bisexual men living with HIV: Wellbeing, connectedness and the impact of stigma. AIDS and Behavior.

[CR68] Qiao S, Zhou G, Li X (2018). Disclosure of same-sex behaviors to health-care providers and uptake of HIV testing for men who have sex with men: A systematic review. American Journal of Men's Health.

[CR69] Reay R, Kisely SR, Looi JCL (2021). Better access: Substantial shift to telehealth for allied mental health services during COVID-19 in Australia. Australian Health Review.

[CR70] Reinius M, Zeluf Andersson G, Svedhem V, Wettergren L, Wiklander M, Eriksson LE (2021). Towards a new understanding of HIV-related stigma in the era of efficient treatment- A qualitative reconceptualization of existing theory. Journal of Advanced Nursing.

[CR71] Rendina HJ, Gamarel KE, Pachankis JE, Ventuneac A, Grov C, Parsons JT (2017). Extending the minority stress model to incorporate HIV-positive gay and bisexual men's experiences: A longitudinal examination of mental health and sexual risk behavior. Annals of Behavioral Medicine.

[CR72] Riffkin, R. (2014). *New record highs in moral acceptability: Premarital sex, embryonic stem cell research, euthanasia growing in acceptance*. https://news.gallup.com/poll/170789/new-record-highs-moral-acceptability.aspx

[CR73] Roth GH, Walker ER, Talley CL, Hussen SA (2022). 'It's a very grey, very messy area': A qualitative examination of factors influencing undetectable gay men's HIV status disclosure to sexual partners. Culture, Health and Sexuality, Advanced Publication..

[CR74] Skerrett DM, Kõlves K, De Leo D (2015). Are LGBT populations at a higher risk for suicidal behaviors in Australia? Research findings and implications. Journal of Homosexuality.

[CR75] Smit, P. J., Brady, M., Carter, M., Fernandes, R., Lamore, L., Meulbroek, M., Ohayon, M., Platteau, T., Rehberg, P., Rockstroh, J. K., & Thompson, M. (2012, 04//). HIV-related stigma within communities of gay men: A literature review [Article]. *AIDS Care, 24*(4), 405–412. 10.1080/09540121.2011.613910 10.1080/09540121.2011.613910 PMC3379736 22117138

[CR76] Stainton A, Chisholm K, Kaiser N, Rosen M, Upthegrove R, Ruhrmann S, Wood SJ (2019). Resilience as a multimodal dynamic process. Early Intervention in Psychiatry.

[CR77] Turan JM, Elafros MA, Logie CH, Banik S, Turan B, Crockett KB, Pescosolido B, Murray SM (2019). Challenges and opportunities in examining and addressing intersectional stigma and health. BMC Medicine.

[CR78] US Department of Health and Human Services. (2018). *A timeline of HIV/AIDS*. https://www.hiv.gov/hiv-basics/overview/history/hiv-and-aids-timeline

[CR79] Verrelli S, White FA, Harvey LJ, Pulciani MR (2019). Minority sress, social support, and the mental health of lesbian, gay, and bisexual Australians during the Australian marriage law postal survey. Australian Psychologist.

[CR80] Walch, S. E., Ngamake, S. T., Bovornusvakool, W., & Walker, S. V. (2016, 2016–03–02). Discrimination, internalized homophobia, and concealment in sexual minority physical and mental health. *Psychology of Sexual Orientation and Gender Diversity, 3*(1), 37–48. 10.1037/sgd0000146

[CR81] Walker, L. (2019). There’s no pill to help you deal with the guilt and shame: Contemporary experiences of HIV in the United Kingdom. *Health (London, England : 1997), 23*(1), 97–113. 10.1177/1363459317739436 10.1177/1363459317739436 29090636

[CR82] Windle G (2011). What is resilience? A review and concept analysis. Reviews in Clinical Gerontology.

[CR83] Ziersch A, Walsh M, Baak M, Rowley G, Oudih E, Mwanri L (2021). “It is not an acceptable disease”: A qualitative study of HIV-related stigma and discrimination and impacts on health and wellbeing for people from ethnically diverse backgrounds in Australia. BMC Public Health.

